# Effect of different sports drink compositions on endurance performance and substrate oxidation: a randomized, double-blind, placebo-controlled crossover study in trained athletes

**DOI:** 10.1080/15502783.2025.2516004

**Published:** 2025-06-04

**Authors:** Sascha Ketelhut, Martin Moehle, Laura Hottenrott

**Affiliations:** a University of Bern, Institute of Sport Science, Bern, Switzerland; b Martin-Luther-University Halle-Wittenberg, Institute of Sport Science, Halle (Saale), Germany; c Martin-Luther-University of Halle-Wittenberg, Institute for Performance Diagnostics and Health Promotion, Halle (Saale), Germany

**Keywords:** Endurance performance, supplementations, carbohydrate, amino acids, beetroot

## Abstract

**Background:**

The consumption of sports drinks before, during, and after endurance exercise is a common practice among athletes. These drinks typically contain a variety of components, each of which has been shown to offer individual benefits. However, the combined effects of these components, as formulated in many sports drinks, have not been thoroughly investigated. This study aims to evaluate the impact of typical sports drink components on endurance performance, perceived exertion, and carbohydrate and fat metabolism, using a sequential additive design.

**Methods:**

Twelve healthy, trained endurance athletes aged 20 to 35 years participated in a randomized, double-blind, placebo-controlled study. The study involved a baseline assessment and four exercise protocols, each separated by a 7-day washout period. During each exercise protocol, participants cycled for 40 minutes at 80% of their previously determined peak oxygen uptake , followed by an incremental protocol performed to voluntary exhaustion. Participants were randomly assigned to ingest one of four 300 ml solutions 60 and 30 minutes before each exercise protocol: solution A (placebo mineral water), solution B (dextrose and sodium), solution C (solution B + beetroot extract), or solution D (solution C + arginine and L-citrulline). Ventilation and heart rate were continuously monitored before and throughout the exercise. Lactate and glucose levels were measured at specific time points before and during the exercise, while ratings of perceived exertion were recorded every 10 minutes. Power output was continuously tracked throughout the exercise protocol. Body weight was assessed both before and after each exercise session.

**Results:**

There were no significant differences between the four solutions in terms of time to exhaustion (*p* = .703), power output (*p* = .822), peak oxygen uptake (*p* = 990), maximum lactate concentration (*p* = .720), and maximum heart rate (*p* = .884). During the exercise, no significant differences were observed in lactate and glucose concentrations, heart rate, or ventilatory parameters (ps > .050). However, significant differences (*p* < .001) in blood glucose concentrations were noted at rest and during the warm-up phase.

**Conclusions:**

The effect of the tested sports drink compositions on performance during the selected exercise protocol to voluntary exhaustion appears minimal. This study found no significant differences between the solutions and the placebo. Thus, it can be concluded that consuming a dextrose-based solution before exercise did not offer any performance advantage over water. The additional substances included in solutions B, C, and D did not influence performance, carbohydrate metabolism, or fat metabolism during the exercise.

## 1. Introduction

The consumption of sports drinks has become standard practice among athletes seeking to enhance their performance and recovery during endurance exercise. These beverages are often marketed with claims of improved performance, reduced fatigue, and faster recovery, owing to their specific formulations. These include varying concentrations of carbohydrates and other ergogenic substances such as sodium, nitrates, beta-alanine, amino acids, and various plant extracts. In the ever-expanding range of products, sports drinks vary significantly in composition, from simple carbohydrate solutions to complex formulations with added performance-enhancing ingredients. However, our understanding of the effects of these components and their various compositions remains limited. This study aims to assess how different compositions of sports drink components influence endurance performance.

Carbohydrates have received significant attention in sports nutrition due to their crucial role in performance and training adaptation [1,2]. They are an essential energy source for the brain and central nervous system and serve as a versatile fuel for muscles, supporting a wide range of exercise intensities through both anaerobic and aerobic pathways [3,4]. In addition to carbohydrates, sodium has been a key component in sports drinks, traditionally used to combat hyponatremia and maintain fluid balance [1,5]. Moreover, when consumed in a pre-exercise dose, sodium has been shown to enhance extracellular buffering capacity and improve performance [6].

Amino acids – particularly arginine and L-citrulline – are commonly used as sports supplements due to their ability to promote vasodilation by increasing nitric oxide production [7,8]. This results in greater delivery of nutrients and oxygen to active tissues, which helps reduce fatigue and enhance performance [9,10]. Moreover, increased blood flow supports protein synthesis and facilitates muscle recovery, thereby further enhancing performance [11] These amino acids have gained significant attention for their potential synergistic effects [12,13]. Evidence suggests that their combined intake may be more effective in enhancing performance outcomes [14,15] as the combination can increase plasma arginine concentrations and rapidly boost nitric oxide bioavailability [8]

More recently, beetroot extract has gained increasing recognition for its health benefits and positive effects on exercise performance, primarily due to its diverse bioactive compounds, particularly inorganic nitrate [16,17]. Inorganic nitrate is notable for its ability to convert into nitric oxide in the body [18]. A recent systematic review with meta-analysis suggests that acute or short-term administration of beetroot extract may be an effective intervention to enhance muscular endurance, especially in isotonic exercises, and muscular strength, particularly in fatigued states, in healthy male individuals [17].

While these individual components have shown performance-enhancing effects, the potential added benefits of combining them, as seen in various sports drinks, remain underexplored. This study aims to address this gap by evaluating these different components in a sequential additive design and investigating their impact on endurance performance, perceived exertion, and metabolic responses related to carbohydrate and fat utilization. To achieve this, four different drink formulations – comprising a placebo, dextrose, dextrose with beetroot extract, and a combination of dextrose, beetroot extract, arginine, and L-citrulline -were tested using a specifically designed exercise protocol that replicated the typical loading patterns encountered during endurance competitions. We hypothesized that the different solutions would have distinct effects on energy metabolism, heart rate, and perceived exertion, with the addition of more components leading to an additive effect. This study seeks to provide insights into the effectiveness of complex sports drinks and their components, contributing to a better understanding of their role in enhancing athletic performance and optimizing endurance exercise outcomes.

## 2. Materials and methods

### 2.1. Participants

Twelve trained male endurance athletes enrolled voluntarily in this study. Participants were included if they met the following inclusion criteria: 1) male; 2) competitive cyclists or triathletes (regularly competing at the national or international level); 3) aged 20 to 45 years; 4) a minimum weekly training volume of ≥6 hours of endurance exercise, including at least 3 hours of cycling. Exclusion criteria were: 1) any acute illness or health condition; 2) orthopedic limitations; 3) use of medication affecting the cardiovascular or digestive system; or 4) a diagnosis of cardiovascular disease.

Participants were recruited from the student population of Martin-Luther-University Halle-Wittenberg, local sports clubs, and athletes regularly undergoing performance diagnostics at our laboratory through e-mails, flyers, social media and word of mouth. An a priori power analysis was conducted using G*Power (Version 3.1.2; Heinrich Heine University, Düsseldorf, Germany). Assuming an effect size of 0.5 for endurance performance [19] with an alpha level of .05, a sample size of 9 participants was determined to be necessary to achieve a power of 0.8. Before enrollment, participants were thoroughly briefed on the study’s objectives and procedures and provided written informed consent. Additionally, they completed the Physical Activity Readiness Questionnaire.

### 2.2. Study design

A randomized, double-blind, placebo-controlled study design was utilized. Participants visited the laboratory on five separate test days, with a 7-day washout period between each session. The sessions were all scheduled for the same time each morning and were conducted in the laboratory at the Institute of Sports Science, Martin Luther University Halle-Wittenberg. Trained study staff conducted all measurements using standardized procedures and the same equipment. The temperature of the lab was controlled at 20 ± 1°C.

The study design included a baseline assessment during the first visit to determine peak oxygen consumption (VO_2_peak). On the subsequent four test days, participants completed a specific exercise protocol while receiving one of four different solutions, administered in a randomized order. Throughout the study period, participants maintained their regular training routines, with no training camps or intensive training blocks permitted. Daily training logs were collected to monitor participants’ training activities.

The study was conducted in accordance with the Declaration of Helsinki and was approved by the institutional ethics committee of the Medical Faculty of Martin-Luther-University Halle-Wittenberg (2018–49, 12 Mai 2018).

### 2.3. Dietary control

Participants were provided with specific dietary guidelines, including a concrete example of a meal composition to follow the evening before the day of testing. The recommended meal was designed to consist of 40% carbohydrates, 40% fat, and 20% protein of total calories consumed, to ensure standardized macronutrient intake prior to testing. On the test day, participants consumed a standardized granola (Dr. Feil Eiweiß-Müsli beere, ultraSPORTS, Daliun LLC, St. Petersburg, USA) mixed with a fixed amount of whole milk yogurt, which was to be eaten 2.5 hours before the start of the test. The participants were instructed to avoid supplements for three days prior to each test day and to abstain from caffeine and carbohydrate-containing beverages on the day of the examination. They were also instructed to maintain their usual diet throughout the study period. Dietary logs were provided to record all food and fluid intake, and these were reviewed by the study team to verify adherence to the dietary protocol.

### 2.4. Baseline assessment

During the first visit, baseline assessments were conducted. Participants completed a medical questionnaire to confirm they were not taking any medications or supplements that could interfere with the study. Body mass was measured using a scale (BC-545N, Tanita Europe BV, Netherlands). Thereafter VO_2_peak was assessed during an incremental exercise test on a high-performance bicycle ergometer (E 2000 s, FES, Berlin, Germany) using a breath-by-breath gas collection system (Metalyzer 3B, Cortex, Leipzig, Germany). A 15-second rolling average filter was applied to the breath-by-breath data, which was then used to determine VO_2_peak. After an 8-minute warm-up, the test began with an initial workload of 100 watts, with increments of 20 watts every 3 minutes until voluntary exhaustion. The pedal cadence was set at 80–90 revolutions per minute.

### 2.5. Solution composition

In a double-blind procedure, each participant received one of the four solutions on the different test days, in randomized order. The solution composition is displayed in Table 1. All solutions were prepared using Quellbrunn natural mineral water (Urstromquelle GmbH & Co. KG, Calcium: 60 mg/l, Magnesium: 6 mg/l, Bicarbonate: 204 mg/l, Potassium: 0 mg/l, Sodium: 4 mg/l, Chloride: 5 mg/l, Sulfate: 0 mg/l, Total mineralization: 279 mg/l). Participants consumed the liquid solutions from identical opaque bottles before the exercise protocols, making it impossible for them to distinguish the solutions by appearance. Furthermore, solutions were matched for volume, aroma, taste, and texture. The distribution and preparation of the solutions were managed by staff who were not involved in the study.

**Table 1.** Solution composition.

Solution (300 ml)	Composition	kcal
Placebo	Natural mineral water with 0.18 g citric acid monohydrate (for a comparable taste)	0
Dextrose drink (Dex)	Placebo with 34 g dextrose and 0.45 g Sodium	140
Dextrose plus drink (Dex+)	Dex with 2.6 g beetroot powder from plant extracts	140
Dextrose plus – amino acid drink (DexAA+)	Dex+ with 2.3 g arginine and 0.7 g citrulline malate	159

Composition and kcal are presented for 300 ml of the solution.

### 2.6. Experimental sessions

Upon arrival, body mass was assessed. Following this, a five-minute resting spirometry measurement was conducted while the participant was seated. The participant then consumed 300 ml of the assigned solutions. After 30 minutes, they consumed an additional 300 ml of the same solution before beginning the exercise protocol. Participants were instructed not to consume anything else, aside from the assigned solution, before or during the testing period.

The exercise protocols were performed on the same bicycle ergometer used during the baseline incremental exercise test. It began with a 30-minute warm-up at 50% VO_2_peak, immediately followed by a 40-minute continuous exercise at 80% VO_2_peak. This was then directly followed by an incremental protocol, with resistance increasing at a rate of 10 watts per minute until voluntary exhaustion. Voluntary exhaustion was defined as the inability to maintain a pedal rate above 60 rpm.

This exercise protocol was used as it simulates the typical demands encountered during endurance competitions. Standardized verbal encouragement (content, frequency) was provided to ensure participants exerted maximal effort. At the end of the exercise protocol, body mass was reassessed. If participants urinated after the first solution intake, the urine volume was recorded and accounted for in the calculation of body mass loss. A 600 ml fluid intake was subtracted from posttest body mass measurements.

### 2.7. Measurements

**Figure 1.**
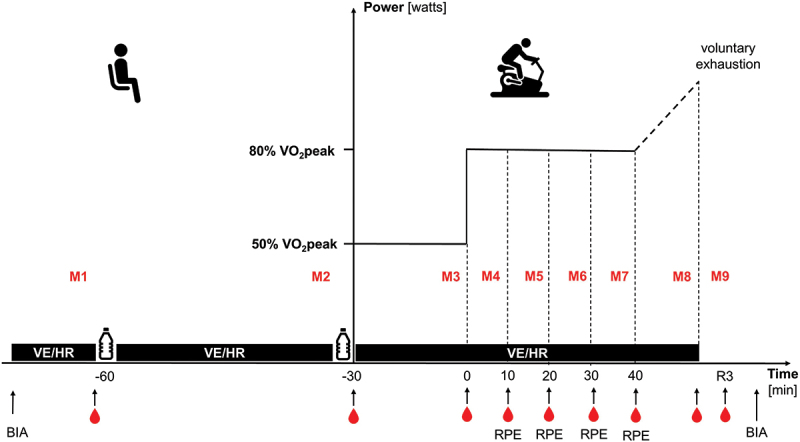
Protocol of experimental sessions.

Body mass was assessed with a scale (BC-545N, Tanita Europe BV, Netherlands). Blood samples were collected from the hyperemic earlobe before the initial consumption of the solutions (M1), before and after the warm-up (M2, M3), every 10 minutes during the exercise protocol (M4-M7), immediately after the exercise (M8), and three minutes post exercise (M9; Figure 1). These samples were analyzed for blood lactate and glucose levels using the Super GL Ambulance (Dr. Müller, Freital, Germany).

The figure illustrates the procedure of the experimental sessions and indicates the measurement time points for various outcomes. *M* = measurement time points; VO_2_peak = peak oxygen consumption; BIA = bioimpedance analysis; $\dot{V}$E = ventilation; HR = heart rate; bottle icon = indicates time of solution intake; drop icon = indicates time of blood samples for lactate and glucose levels.

Ventilation was recorded for five minutes at rest before solution consumption and then continuously until the end of the exercise using a breath-by-breath gas collection system (Metalyzer 3B, Cortex, Leipzig, Germany). The measurement was paused for two minutes during solution intake. For the rest measurements (M1 and M2) and the warm-up (M3), the mean values of the last two minutes of each phase were calculated. For the 40-minute continuous exercise at 80% of VO_2_peak, mean values were computed for minutes 2 to 10 (M4), minutes 10 to 20 (M5), minutes 20 to 30 (M6), and minutes 30 to 40 (M7). For the subsequent incremental phase, the mean value of the last 30 seconds before test cessation was determined (M8).

Heart rate was monitored continuously using a chest strap and heart rate monitor (H7 and RS 800CX, Polar Electro, Kempele, Finland). Data were recorded in 5-second intervals, and mean heart rate values for the respective measurement time points were calculated. Rating of perceived exertion was conducted every 10 minutes during the continuous exercise at 80% VO_2_peak (M4 to M7) using the Borg-RPE scale [20]. This interval allowed a sufficient temporal resolution to detect trends in exertion without introducing excessive disruption to the participants’ exercise flow. Time to exhaustion was calculated as the duration of the 40-minute continuous exercise plus the time completed during the incremental protocol. After completing the exercise protocol, participants were asked if they had experienced any gastrointestinal side effects, such as nausea, bloating, or discomfort.

### 2.8. Statistical analysis

Data were analyzed using SPSS version 26.0 (IBM Corp., Armonk, NY, USA). Normality of the data was assessed using the Shapiro-Wilk test. For within-group comparisons, a two-tailed paired t-test was performed for normally distributed data, while the Wilcoxon signed-rank test was used for non-normally distributed data. Differences in outcomes between the four solutions were evaluated with a one-way analysis of variance (ANOVA). To assess interaction effects (time × condition) a series of repeated-measures analysis of variance (ANOVA) were calculated with Bonferroni corrected post hoc tests. Partial eta-squared (*η*^*2*^_*p*_) values were calculated to estimate the effect sizes (small effect: *η*^*2*^_*p*_ = .014, medium effect: *η*^*2*^_*p*_ = .06, large effect: *η*^*2*^_*p*_ = .14) for the interactions [21]. Statistical significance was set at *p* < .05.

## 3. Results

Two participants became ill for reasons unrelated to the study and were therefore excluded. The remaining 10 participants successfully completed all four trials. Participant characteristics are presented in Table 2. According to body mass index, three participants were classified as being overweight. Based on VO_2_peak cutoff values [22] eight participants were classified as superior and two as good. None of the participants experienced gastrointestinal intolerance following the consumption of the four different solutions.

**Table 2.** Participant characteristics.

Outcome	
Age (years)	33.6 ± 8.3
Body height (cm)	180.8 ± 7.9
Body mass (kg)	79.0 ± 11.7
Body Mass Index (kg/m^2^)	24.1 ± 2.7
VO_2_peak (mL/kg/min)	51.56 ± 7.33
Maximal Power output (Watts)	299.0 ± 32.9

### 3.1. Maximal performance outcomes

No significant changes in maximal power output (*p* = .822) or VO_2_peak (*p* = .673) were observed over the four-week period. Participants achieved an average of 292.0 ± 21.9 watts and 49.07 ± 6.95 mL/min/kg in the first trial, compared to 306.4 ± 42.6 watts and 49.92 ± 8.99 mL/min/kg in the fourth trial. Similarly, there were no significant changes in time to exhaustion (*p* = .703), maximal lactate concentration (*p* = .868), or maximal heart rate (*p* = .884).

**Figure 2.**
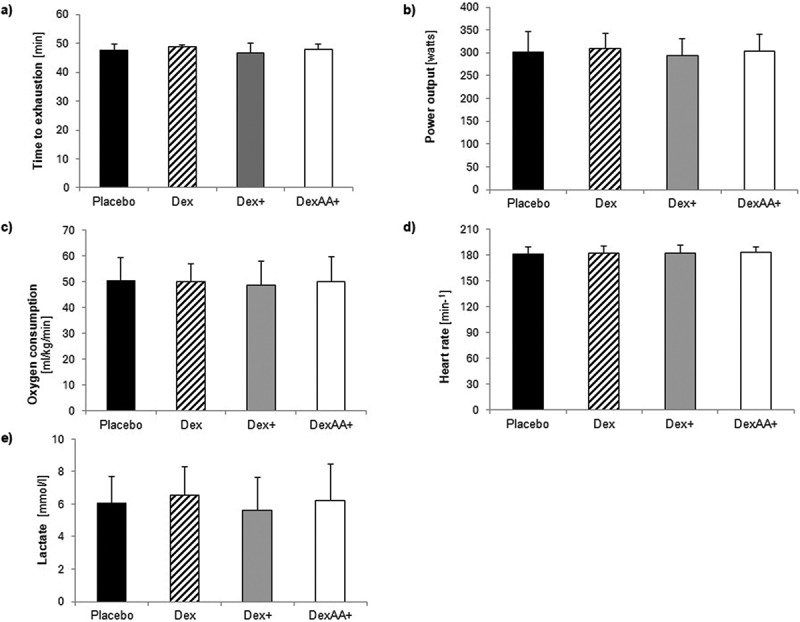
Effects of the different solutions on maximal performance outcomes during the time trials.

The ANOVAs revealed no significant differences in time to exhaustion (*p* = .703, *η*^2^_*p*_ = .038, Figure 2(a)), maximal power output (*p* = .822, *η*^2^_*p*_ = .025, Figure 2(b)), maximal oxygen uptake (*p* = .990, *η*^2^_*p*_ = .003, Figure 2(c)), maximal heart rate (*p* = .884, *η*^2^_*p*_ = .018, Figure 2(d)) or maximal lactate concentration (*p* = .720, *η*^2^_*p*_ = .036, Figure 2(e)) during the exercise protocol for the different conditions (Placebo, Dex, Dex+, DexAA+).

Dex= dextrose drink; Dex+= Dextrose plus beet root powder, DexAA+= dextrose plus beet root powder and amino acid

The time to exhaustion and power output for each condition were as follows: 47.5 ± 2.4 minutes and 301.6 ± 44.9 watts for the placebo, 48.7 ± 0.7 minutes and 309.3 ± 33.5 watts for Dex, 46.7 ± 3.4 minutes and 292.8 ± 38.6 watts for Dex+, and 47.9 ± 1.8 minutes and 303.3 ± 36.9 watts for DexAA+. Maximal lactate concentrations were 6.03 ± 1.68 mmol/l for the placebo, 6.55 ± 1.75 mmol/l for Dex, 5.6 ± 2.0 mmol/l for Dex+, and 6.21 ± 2.25 mmol/l for DexAA+. Maximal heart rates were 181.2 ± 8.8 min^−1^ for the Placebo, 182.6 ± 8.5 min^−1^ for Dex, 183.0 ± 8.5 min^−1^ for Dex+, and 183.1 ± 6.5 min^−1^ for DexAA+.

### 3.2. Trends in outcomes for different solutions

**Figure 3.**
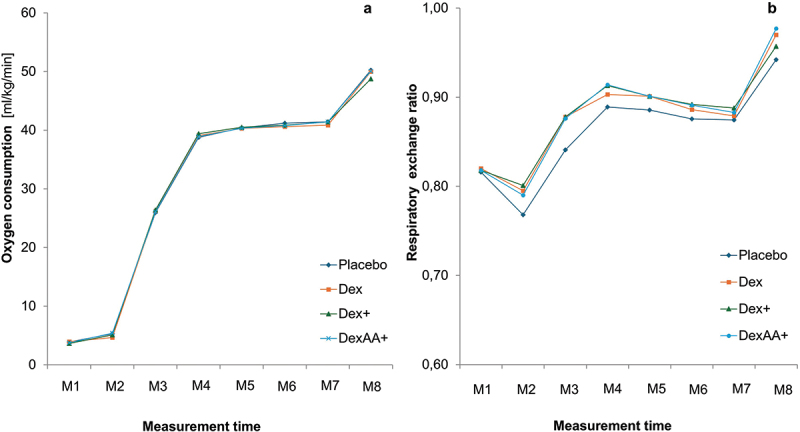
Trends in ventilatory outcomes for different solutions.

**Figure 4.**
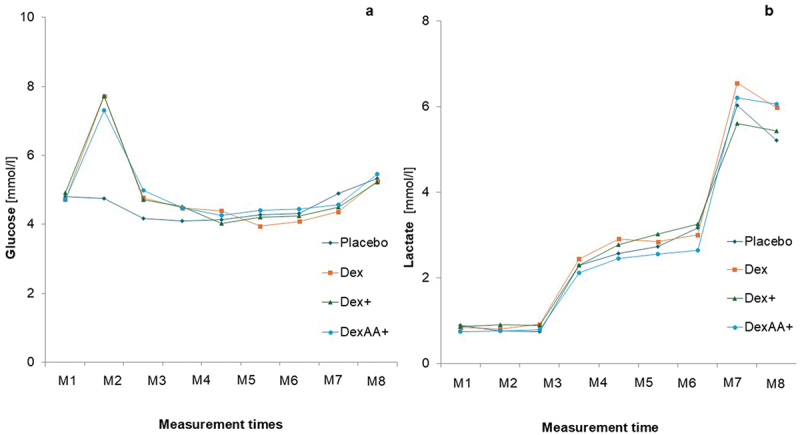
Trends in metabolic substrates for different solutions.

**Figure 5.**
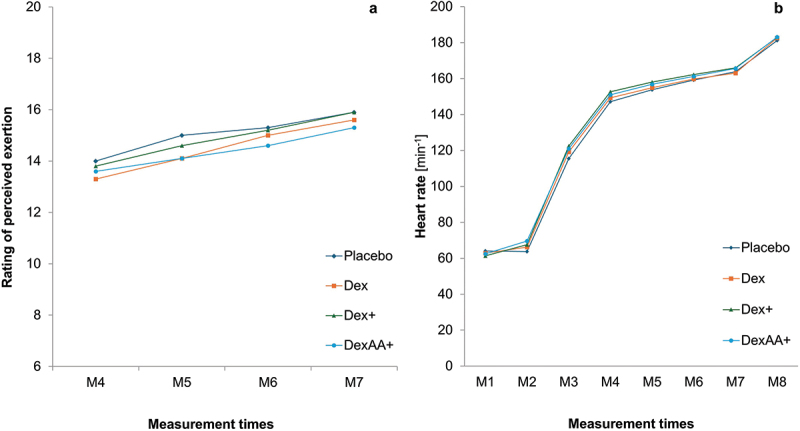
Trends in rating of perceived exertion and heart rate for different solutions.

Figures 3–5 display the trends of outcomes at the different measurement points across the four time trials. No significant time × condition interactions could be detected for oxygen consumption (*p* = .995; *η*^2^_*p*_ =.007, Figure 3(a)), respiratory exchange ratio (*p* = .774; *η*^2^_*p*_ = .050, Figure 3(b)), lactate concentration (*p* = .842; *η*^2^_*p*_ = .037, Figure 4(b)), perceived exertion (*p* = .717, *η*^2^_*p*_ = .047, Figure 5(a) and heart rate (*p* = .813, *η*^2^_*p*_ = .043, Figure 5(b)). However, a significant interaction effect (*p* < .001, *η*^2^_*p*_ = .311) could be detected for glucose concentration (Figure 4(a)). Glucose concentrations significantly increased 30 minutes after consumption (M2) of Dex (*p* = .002), Dex+ (*p* < .001), and DexAA+ (*p* < .001), but not after consuming the placebo (*p* = .787) (Figure 3(d)). After the warm-up (M3), a significant decrease in glucose levels was observed compared to M2 for all conditions (placebo: *p* = .022, Dex: *p* = .005, Dex+: *p* < .001, DexAA+: *p* < .001). Glucose levels remained relatively stable at subsequent measurement points, with no significant changes until the recovery phase (R3), during which glucose levels significantly increased across all conditions (*p* < .001).

*M*= Measurement time points. Dex= dextrose drink; Dex+= Dextrose plus beet root powder, DexAA+= dextrose plus beet root powder and amino acid

*M*= Measurement time points. Dex= dextrose drink; Dex+= Dextrose plus beet root powder, DexAA+= dextrose plus beet root powder and amino acid

*M*= Measurement time points. Dex= dextrose drink; Dex+= Dextrose plus beet root powder, DexAA+= dextrose plus beet root powder and amino acid

### 3.3. Pre-post comparison

**Figure 6.**
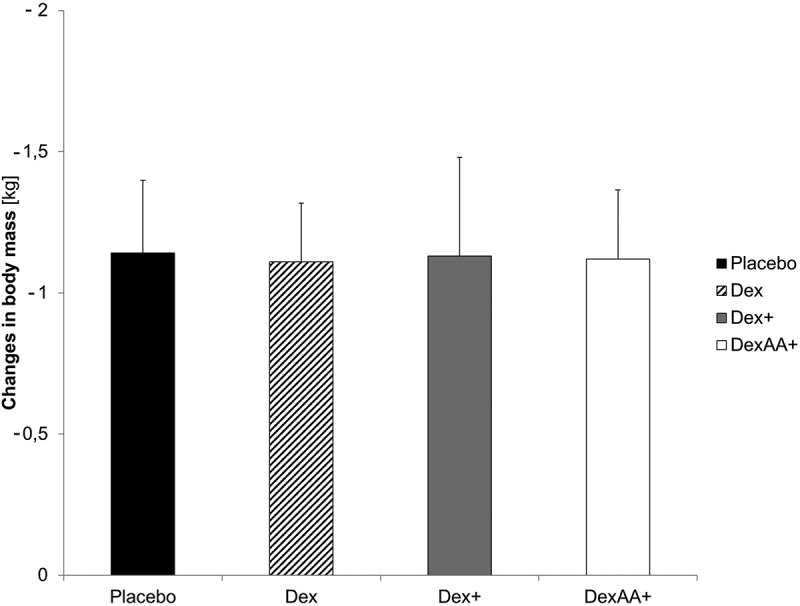
Changes in body mass before and after the time trials for different solutions.

There was a significant reduction in body mass across all time trials (*p* < .001, *η*^2^_*p*_ = .850), with an average decrease of 1.25 ± 0.26 kg. No significant differences were observed between the conditions (*p* = .755, *η*^2^_*p*_ = .032) (Figure 6).

## 4. Discussion

This study aimed to assess the impact of various sports drink components on endurance performance, perceived exertion, and metabolic responses related to carbohydrate and fat utilization. The findings indicate that pre-exercise supplementation offered no significant performance advantages over a placebo, nor did it influence carbohydrate or fat metabolism or perceived exertion during the exercise protocol.

### 4.1. Maximal performance outcomes

While previous studies indicate that carbohydrate consumption can enhance performance [4], there is evidence for limited performance improvement in athletes with well-replenished glycogen stores when consuming carbohydrate-rich solution shortly before endurance exercise [23,24]. Due to the stringent dietary control and restrictions on training volume and intensity before the test days, it is likely that the participants had adequately replenished glycogen stores. This may explain why there were no significant performance differences between the carbohydrate-rich solutions (Dex, Dex+, DexAA+) and the placebo. Additionally, it can be argued that the exercise duration of the protocol was too short, as glycogen stores in the muscles and liver are typically sufficient for exercise lasting up to 60 minutes [4,23,25]. This is supported by the observation that glucose levels during the exercise did not differ significantly from those observed with the placebo.

Adding beetroot extract in the Dex+ solution had no significant impact on performance outcomes. This conflicts with previous studies suggesting that beetroot extract can enhance endurance performance by promoting vasodilation through its nitrate content [17]. It could be argued that the acute supplementation in this study was not at a high enough dosage to yield noticeable improvements. This assumption is supported by findings from Jones [26] and Cermak et al [27], who suggests that an acute beetroot/nitrate supplementation has none or only very limited ergogenic potential. Additionally, the ergogenic effect of nitrate is influenced by factors such as individual fitness level, age, muscle fiber type, and the duration and intensity of exercise [26]. Notably, highly trained individuals, such as those in the present study, tend to show no positive effects from nitrate supplementation [28].

While the literature supports the performance-enhancing effects of arginine [29], both as a nitrate booster and through its influence on various metabolic processes [9,10], these effects were not observed in the present study. The DexAA+ solution had no effects on the performance outcomes. A possible explanation for this may be the relatively low doses of arginine (2.3 g) and L-citrulline (0.7 g) used in the study. According to the meta-analysis by Viribay et al. [29], arginine supplementation at 0.15 g/kg of body mass, ingested 60–90 minutes before exercise, is necessary to improve both anaerobic and aerobic performance outcomes.

### 4.2. Trends in outcomes for different solutions

The different solutions had no impact on the progression of lactate concentration. No significant differences were found between the solutions at any of the nine measurement points, nor were there differences in the maximal lactate concentration at the end of the incremental protocol. With the direct availability of exogenous carbohydrates, higher lactate values would have been expected. It is likely that the endogenous energy stores were sufficiently replenished for the required exercise, as discussed earlier.

Similarly, no significant differences in heart rate were observed between the conditions. Given that no performance differences were found between the conditions, it is reasonable that no differences in heart rate were observed during the exercise. Similar results were reported by Levine et al. [30], who found no differences in heart rate during exercise when comparing glucose, fructose, and water intake 45 minutes before cycling.

Significant differences in blood glucose concentration were observed at measurement point 2, following the second intake, between the placebo and the three solutions. This aligns with findings by Levine et al. [30], who reported a significant increase in blood glucose levels at rest 45 minutes after administering 75 g of glucose, accompanied by a marked rise in insulin and a trend toward decreased glycerol levels. Although this study used only 34 g of dextrose, a significant increase in blood glucose was still observed 30 minutes post-ingestion. No differences in blood glucose concentrations were found among the three solutions (Dex, Dex+, and DexAA+), either at rest or during exercise. The energy from the dextrose solution (Dex, Dex+, and DexAA+) did not result in significant changes in blood glucose levels during exercise compared to the placebo. This could be due to the relatively short exercise duration of 60 minutes, during which the endogenous glycogen stores in the muscles and liver are typically sufficient to maintain blood glucose levels [4,23,25].

The supplementation of beetroot extract (Dex+ and DexAA+) did not show differences compared to the Dex solution (without beetroot) with respect to lactate or glucose levels. This is in line with Cermak et al. [31], who found no differences in lactate, glucose, or insulin concentrations in a cycle ergometer test following six days of beetroot juice supplementation (8 mmol nitrate per day) compared to the placebo drink. The total amount of nitrate we administered (9.6 mmol) was very similar to the daily nitrate dose used in their study.

No differences in blood glucose concentration were observed between Dex+ and DexAA+. Consequently, this study did not confirm the beneficial effects of arginine supplementation on blood glucose levels as suggested in previous research [32]. Contrary to our assumption, there were no significant differences in the respiratory exchange ratio between the four conditions, which contradicts findings from previous studies [33] that involved carbohydrate intake during exercise (e.g. every 15–30 minutes). While slight differences were observed between the placebo and the other solutions during certain time points (M2-M6, and M9), these differences were not statistically significant. Possibly, a larger sample size might have provided more conclusive results. Additionally, the two doses of 34 g dextrose before exercise may have been insufficient to affect the CO_2_/O_2_ ratio. However, when examining CO_2_ output alone, significant differences were observed between the placebo and the other solutions at time point M3, likely due to increased carbohydrate metabolism from the supplements.

The different conditions did not affect perceived exertion measured at four time points during the 40-minute endurance test. Since no differences were observed in physiological, metabolic, or cardiac parameters across the conditions, it is unsurprising that perceived exertion also showed no variation between groups. These findings are consistent with those of Rothschild et al. [34], who reported no effect of pre-exercise carbohydrate ingestion on perceived exertion during submaximal exercise in trained cyclists. In contrast, Burgess et al. [33] reported higher perceived exertion with water compared to glucose intake every 15 minutes during an intense 180-minute exercise session. However, these differences only became significant after 60 minutes of exercise.

Throughout the exercise sessions, there was a significant reduction in body mass across all conditions, likely attributable to fluid loss from sweating. However, no significant differences were observed between conditions.

### 4.3. Limitations

There are several methodological limitations that should be considered when interpreting the results. First, due to the dropout of two participants, the ideal distribution of the four conditions across the trial days was not fully achieved. Second, the study exclusively enrolled healthy, highly trained male participants, limiting the generalizability of the findings to other populations. Third, despite the applied inclusion and exclusion criteria, the participant group was not entirely homogenous. A more uniform and better trained sample might have revealed clearer effects. Fourth, participants’ hydration status was not controlled prior to testing sessions, which may have introduced variability in body mass measurements. Lastly, the study used fixed (absolute) doses of ingredients, which may not account for individual differences in body size and composition. Future studies may consider using weight-adjusted dosing to explore how body weight influences the response to supplementation.

## 5. Conclusion

Finding the optimal sports drink and determining the best timing for its intake are common concerns among athletes. This study showed that consuming a dextrose-based solution before exercise did not offer any performance advantage over water in highly trained males. Additionally, adding beetroot extract, or beetroot extract combined with amino acids had no measurable impact on performance, carbohydrate or fat metabolism, or perceived exertion during the exercise sessions. The lack of significant differences compared to a placebo suggests that well-fed athletes do not benefit from supplements containing carbohydrates, beetroot extract, and amino acids during 40–50 minutes of exercise at 20°C. Future research should explore whether athletes with depleted glycogen stores might experience greater benefits, as well as the potential effects of higher supplementation dosages or different timing of intake.
